# The effect of *Phyllanthus emblica* (Amla) fruit supplementation on the rumen microbiota and its correlation with rumen fermentation in dairy cows

**DOI:** 10.3389/fmicb.2024.1365681

**Published:** 2024-05-13

**Authors:** Mekonnen Tilahun, Lu Ma, Todd R. Callaway, Jianchu Xu, Dengpan Bu

**Affiliations:** ^1^State Key Laboratory of Animal Nutrition and Feeding, Institute of Animal Science, Chinese Academy of Agricultural Sciences, Beijing, China; ^2^Yunnan Key Laboratory for Wild Plant Resources, Department of Economic Plants and Biotechnology, Kunming Institute of Botany, Chinese Academy of Sciences, Kunming, China; ^3^Department of Animal and Dairy Science, University of Georgia, Athens, GA, United States; ^4^World Agroforestry Centre East and Central Asia, Kunming, China; ^5^CAAS-ICRAF Joint Lab on Agroforestry and Sustainable Animal Husbandry, Beijing, China

**Keywords:** Amla fruit, rumen microbiota, *Oscillospiraceae*, biohydrogenation, *Butyrivibrio*, dairy cows

## Abstract

**Introduction:**

Medicinal plants, rich in phytochemicals like phenolic acids, flavonoids, and tannins, offer potential benefits in enhancing productivity, quality, and animal health. Amla fruit (*Phyllanthus emblica*) is one such plant with promising attributes. This study aimed to investigate the impact of fresh Amla fruit (FAF) supplementation on ruminal microbial composition and its correlation with rumen fermentation in lactating dairy cows.

**Methods:**

The study employed a repeated crossover design involving eight ruminally cannulated mid-lactation Holstein dairy cows. Animals received varying levels of fresh Amla fruit supplementation (0, 200, 400, and 600 g/d).

**Results:**

When 400 g/d of FAF was added to the diet, there was a significant increase in the relative abundance of Firmicutes (*p* = 0.02). However, at 200 g/d, the relative abundance of ruminal Bacteroidota was higher than the 0 and 400 g/d FAF supplementation (*p* < 0.01). LEfSe analysis identified distinct taxa, such as *Clostridia vadinBB60* in the 200 g/d group, *Oscillospiraceae* in the 400 g/d group, and *Elusimicrobium* in the 600 g/d group. Notably, the random forest species abundance statistics identified *Oscillospiraceae V9D2013* as a biomarker related to milk yield. *Oscillospiraceae, Bacilli RF39, norank_f Prevotellaceae*, and *Bifidobacterium* were positively correlated with ruminal total VFA and molar proportion of propionate, while *Rikenellaceae RC9* gut group and *Clostridia vadinBB60* were negatively correlated.

**Discussion:**

FAF supplementation affects the abundance of beneficial microbes in a dose-dependent manner, which can improve milk yield, efficiency, rumen health, desirable fatty acids, and animal health.

## 1 Introduction

Feed accounts for a significant proportion of livestock and poultry production costs, ranging from 60 to 70% in most years (Lawrence et al., 2008). However, the growing human population and increased urbanization exert pressure on feed production along with other natural resources, leading to a competition between food and feed production (Lal, 2020). The need to ensure a feed supply from alternative sources has led researchers to explore the use of non-conventional and novel plant sources (Achilonu et al., 2018). However, the feasibility of these alternative feedstuffs depends on various factors, including feed value, animal production responses, and cost compared with conventional feeds (Halmemies-Beauchet-Filleau et al., 2018). Thus, low-input feeding strategies using grain alternatives, such as shrubs, bushes, new forages, insects, and many byproducts of agro-industrial production (e.g., distiller’s grains) are being widely investigated or utilized (Devendra, 1992; Mirzaei-Aghsaghali and Maheri-Sis, 2008; Makkar et al., 2014; Moorby and Fraser, 2021).

Plant secondary metabolites (PSMs) are used as natural additives to animal feed to enhance performance, including improved rumen fermentation efficiency, protein metabolism, methane production, antimicrobial properties, and overall animal health and productivity (Makkar et al., 2009; Grazziotin et al., 2020; Ku-Vera et al., 2020; Kholif et al., 2021). Commercially available PSMs, including tannins, saponins, and essential oils, have the potential to modify the composition and number of ruminal microorganisms, methanogenesis, and biohydrogenation of fatty acids (Patra and Saxena, 2010; Vasta et al., 2019). Although PSMs have a positive impact on animal health and productivity, they often have diverse and contradictory influences on animals, depending on factors such as extracts, dosages, and the type and quality of baseline diets and sometimes difficult to explain (Martínez-Fernández et al., 2013; Cieslak et al., 2014; Machado et al., 2016).

This study investigates the potential of *Phyllanthus emblica* commonly called Amla fruit as a feed additive source for dairy cows. Amla is a fruit belonging to the Euphorbia family and has long been traditionally used for medicinal purposes. It is grown extensively in tropical and subtropical regions (Nisar et al., 2018). Amla (*Phyllanthus emblica*) fruits are rich in various PSMs such as phenolic acids, flavonoids, tannins, alkaloids, glycosides, terpenoids, lipids, and amino acids (Liu et al., 2008; Saini et al., 2022; Tilahun et al., 2022a). In our accompanying study, we identified five primary metabolites in fresh Amla fruit using UPLC-ESI-MS/MS, which were phenolic acids (22%), flavonoids (20%), lipids (20%), amino acids and derivatives (9%), and tannins (7%) (Tilahun et al., 2022b). Furthermore, fruit juice of Amla fruit contains the highest concentration of vitamin C (478.56 mg/100 mL), which is higher than that found in oranges, tangerines, and lemons (Jain and Khurdiya, 2004).

All of the above bioactive components make Amla a fruit with significant medicinal properties. The Amla fruit is known for its numerous health benefits, which include antimicrobial, antioxidant, antiinflammatory, cardioprotective, gastroprotective, neuroprotective, anticancer, and anti-diabetic properties (Saini et al., 2022). However, its bitterness and astringency can significantly hinder its use in food products (Zhang et al., 2024), leading to substantial production waste (Yadav et al., 2020). As such, our research aims to investigate the potential feed value of Amla for dairy cows.

In a companion study (Tilahun et al., 2022a,b), the inclusion of fresh Amla fruit in dairy cow diets affected protozoa, acetate, propionate, ammonia-N concentration, milk nitrogen efficiency, milk yield, desirable milk fatty acid, and antioxidant capacity. In addition, in our laboratory work, the supplementation of Amla fruit powder at 5 g per day improved the antioxidant capacity and immune response of preweaning dairy calves (Nguse et al., 2022). Research findings suggest that the rumen bacterial population is associated with production performance in dairy cows, including milk quality and feed efficiency (Wang G. et al., 2023). Our previous research on dairy cow performance has prompted us to investigate further the potential effects of Amla fruit on rumen microbes that may contribute to performance enhancement at varying dose levels. However, the underlying mechanisms of these effects have not yet been sufficiently explored.

We hypothesized that the inclusion of fresh Amla fruit in the diet would impact ruminal bacterial populations, altering fermentation pathways, protein metabolism, and fatty acid biohydrogenation and impacting animal health. This investigation holds significant potential for understanding rumen microbiota and its association with rumen fermentation in dairy cows. Our objective was to identify bacterial genera that exhibit differential correlation with varying levels of fresh Amla fruit intake, offering innovative insights into its potential utilization as a ruminant feed additive. These research findings hold substantial promise for enriching the existing understanding of the impact of Amla fruit on ruminant nutrition, thus offering valuable implications for advancing livestock industry practices.

## 2 Materials and methods

### 2.1 Animals, diets, and experimental design

The present study adhered to the IAS and CAAS guidelines, as documented in reference IAS20180115. Cow feeding and management procedures and sample collection were previously described (Tilahun et al., 2022a,b). In brief, eight mid-lactation Chinese Holstein dairy cows with ruminant cannulas were used. In this study, cows ranged in parity from 1 to 4, a body weight of 634 ± 67.6 kg, and were 102.1 ± 4.49 days in milk, with a milk yield of 19.4 ± 2.59 kg per day. Using a repeated crossover design, eight lactating cows with cannulas were randomly assigned into two treatment groups based on their milk yield, parity, and body weight. The treatment groups included a control group that was fed a total mixed ration (TMR) without Amla fruit (0 g/d) and treatment group—TMR supplemented with fresh Amla fruit (FAF) in a sequential order at three dose levels (200, 400, or 600 g/d, as-fed basis), which is administered as a top dress. Fresh Amla fruit was added directly to the TMR and given as a top dressing, with half of the daily allowance provided each at 07:30 a.m. and 15:30 p.m.

The TMR diet used for all groups was formulated using AMTS Cattle Pro version 4.14 (2018, AMTS LLC, Groton, NY, United States). After a 14-day adaptation phase, we began Period 1 of the feeding experiment. During Period 1, four cows from the first group were fed the control diet with supplementary FAF feed doses added sequentially (200, 400, and 600 g/d). Each dose was supplemented for 14 days before increasing to the next dose, while the remaining four cows were fed the control diet. Following a 14-day washout period, we began Period 2, during which the control and supplemented groups were exchanged, as previously done in studies conducted by Wang et al. (2018) and Drackley et al. (2007). The diets’ chemical composition, fresh Amla fruit plant secondary metabolites, and experimental layout used in this experiment are extensively reported in the study by Tilahun et al. (2022a,b) and presented in Supplementary Tables S1, S2.

### 2.2 Rumen sample collection and analysis

During each experimental period, rumen fluid samples were collected twice from the cannulated cows on d 12 and 14. Samples were taken three times after morning feeding at 0, 4, and 8 h, to measure various ruminal fermentation characteristics, such as pH, ammonia-nitrogen (NH_3_-N), and volatile fatty acid (VFA) levels. To examine the ruminal microbiota, ruminal contents were collected 4 h after feeding and was sub-sampled (~50 g) before being stored at −20°C.

This study involved the analysis of 112 rumen samples. In the initial stage, 16 samples were taken from eight cannulated cows during their adaptation phase, which were excluded from the subsequent analysis. During period 1, eight cows were divided into two groups, namely the treatment and control groups. From the treatment group in period 1, eight samples were obtained from each FAF dose level; 24 rumen samples were collected from the treatment group from the first group of four cows. In period 1, the same sample size (*n* = 24) was also collected from the other four cows in the control group. After the washout period, the groups were switched, and during period 2, 24 rumen samples were collected from each group, i.e., control (*n* = 24) and treatment (*n* = 24), for microbial analysis. Overall, 96 rumen samples were analyzed, of which 48 were collected from the control and 48 from the treatment groups.

### 2.3 DNA extraction and PCR amplification

Microbial community genomic DNA was extracted from 112 samples using the E.Z.N.A.^®^ soil DNA Kit (Omega Bio-tek, Norcross, GA, United States), following the manufacturer’s guidelines. Extracted DNA was verified for purity on a 1% agarose gel, while the concentration was measured using a NanoDrop 2000 UV–VIS spectrophotometer (Thermo Scientific, Wilmington, United States). The V3-V4 hypervariable region of the bacterial 16S rRNA gene was amplified by the primer pairs 338F (5’-ACTCCTACGGGAGGCAGCAG-3′) and 806R (5’-GGACTACHVGGGTWTCTAAT-3′) using an ABI GeneAmp^®^ 9,700 PCR thermocycler (ABI, CA, United States). The PCR protocol for the amplification of the 16S rRNA gene involved an initial denaturation at 95°C for 3 min, 27 cycles of denaturation at 95°C for 30 s, annealing at 55°C for 30 s, and extension at 72°C for 45 s. The reaction was concluded with a single extension at 72°C for 10 min, stopping at 4°C. The PCR mix contained 4 μL of 5 × TransStart FastPfu buffer, 2 μL of 2.5 mM dNTPs, 0.8 μL of forward primer (5 μM), 0.8 μL of reverse primer (5 μM), 0.4 μL of TransStart FastPfu DNA Polymerase, 10 ng of template DNA, and ddH2O up to 20 μL. The PCR reactions were performed in triplicate, and the PCR product was purified using the AxyPrep DNA Gel Extraction Kit (Axygen Biosciences, Union City, CA, United States), following the manufacturer’s guidelines. Finally, the purified product was quantified using the Quantus™ Fluorometer (Promega, United States).

### 2.4 Illumina MiSeq sequencing

After amplicon purification, they were blended in equal proportions and underwent paired-end sequencing using an Illumina MiSeq PE300 platform (Illumina in San Diego, United States). The standard protocols of Majorbio Bio-Pharm Technology Co., Ltd. in Shanghai, China, were followed.

### 2.5 Data analyses

All data analyses were performed using the Majorbio platform at http://www.majorbio.com. First, the reads were separated according to their barcodes and primers. The sequencing reads underwent quality filtration using FastP version 0.19.6. Subsequently, reads with an average quality score of less than 20 over a 50 bp sliding window were excluded, while reads shorter than 50 bp were truncated. The established protocol ensures that the reads obtained are of high quality, which is paramount for their suitability in the downstream analysis. After removing reads containing ambiguous characteristics, the remaining reads were merged using FLASH version 1.2.11. Random forest model analysis was performed on the Majorbio Cloud Platform with default settings. Additionally, 10-fold stratified K-Folds cross-validation was used to generate receiver operating characteristic (ROC) curves. Overlapping sequences with a length greater than 10 bp were assembled based on their overlapped sequence, with an allowed maximum mismatch ratio of overlap region set at 0.2. Reads that could not be assembled were excluded. Next, operational taxonomic units (OTUs) were clustered using UPARSE version 7.0.1090 with a 97% similarity cutoff, and chimeric sequences were removed during the clustering process. The representative sequence of each OTU underwent an in-depth analysis using RDP Classifier version 2.11 against the 16S rRNA database (silva138/16s_bacteria) with a confidence threshold of 0.7 to determine the diversity of the rumen microbiota.

To compare species composition among different treatment groups at the phylum and genus levels, the Wilcoxon rank-sum test and multiple test corrections (fdr) were utilized. The analysis of variance (ANOSIM/Adonis) from R’s vegan package with unweighted Unifrac dissimilarities was used to test for differences in overall microbiome composition. The significant differences in microbial composition between the four treatment group samples were determined using linear discriminant analysis (LDA) effect size (LEfSe) with a threshold logarithmic LDA score of 2.0. Finally, the Spearman rank correlation coefficient was used to perform a correlation heatmap analysis between environmental factors and selected species. The obtained numerical matrix was visually displayed using a Heatmap diagram through R software (version 3.3.1) (pheatmap package), to analyze the rumen microbiota comprehensively. After eliminating any reads that contained ambiguous characteristics, the remaining reads were merged using FLASH version 1.2.11. Only overlapping sequences that were longer than 10 bp were assembled based on their overlapped sequence, and a maximum allowed mismatch ratio of overlap region was set at 0.2. Any reads that could not be assembled were discarded. Next, operational taxonomic units (OTUs) were clustered using UPARSE version 7.0.1090 with a 97% similarity cutoff. Chimeric sequences were identified and removed during the clustering process.

The representative sequence of each operational taxonomic unit (OTU) underwent an in-depth analysis using RDP Classifier version 2.11 against the 16S rRNA database (silva138/16s_bacteria) with a set confidence threshold of 0.7. To analyze the diversity of the rumen microbiota, Mothur software (version 1.30.2) was used with the alpha diversity index (Sobs, Shannon). To compare species composition among different treatment groups at the phylum and genus levels, the Wilcoxon rank-sum test and multiple test corrections (fdr) were utilized. To test for differences in overall microbiome composition, analysis of variance (ANOSIM/Adonis) from R’s vegan package with unweighted Unifrac dissimilarities was used. Finally, the Spearman rank correlation coefficient was used for a correlation heatmap analysis between environmental factors and selected species. The obtained numerical matrix was visually displayed using a Heatmap diagram through R software (version 3.3.1) (pheatmap package). The alpha microbial diversity estimators were analyzed using PROC MIXED in SAS. The least-squares means statement with the PDIFF option was used to compare means, and polynomial contrasts (linear, quadratic, and cubic) were used to assess the impact of FAF doses, and their contrasts are tabulated. Statistical significance was declared at *p* ≤ 0.05 and trends were identified at 0.05 < *p* ≤ 0.10.

## 3 Results

### 3.1 Effect of dietary fresh Amla fruit supplementation on the diversity of rumen bacterial community

Alpha diversity indices of the bacterial community in the rumen are presented in Table 1. Amla fruit supplementation at 400 g/d demonstrated a cubic effect, reducing the observed bacteria richness (Sobs, *p* = 0.05) and a trend for coverage estimator (ACE, *p* = 0.07) for species richness. Nevertheless, community evenness (Shannon and Simpson indices) did not exhibit any trend with FAF supplementation. The sequencing amount of each sample accurately reflected the diversity of bacterial community types and structures, as indicated by the Good’s coverage of all groups (> 99.2%). The β diversity (Adonis) demonstrated differences between the treatments at the phylum (*R*^2^ = 0.07, *p* = 0.042) and genus (*R*^2^ = 0.05, *p* = 0.038) levels.

**Table 1:** Effect of Amla fruit supplementation at 0, 200, 400, or 600 g/d on alpha microbial diversity estimators of rumen microbial populations from lactating Holstein cows (n = 8).

Estimators	Fresh Amla fruit supplementation g/d				*p*-value			
	0	200	400	600	Dose	Lin	Quad	Cubic
Sobs*	812.98 ± 25.93	907.19 ± 43.49	795.87 ± 44.92	874.75 ± 43.49	0.20	0.66	0.86	0.05
ACE**	1005.9 ± 32.4	1131.7 ± 54.3	1013.9 ± 56.1	1091.9 ± 54.3	0.18	0.50	0.64	0.07
Shannon	4.58 ± 0.06	4.68 ± 0.11	4.52 ± 0.10	4.62 ± 0.10	0.75	0.89	0.99	0.27
Simpson	0.036 ± 0.003	0.042 ± 0.006	0.037 ± 0.006	0.036 ± 0.006	0.83	0.80	0.52	0.51
Coverage	0.993 ± 0.00	0.992 ± 0.00	0.993 ± 0.00	0.992 ± 0.00	0.17	0.29	0.50	0.15

*The observed richness (Sobs), **abundance-based coverage estimator (ACE). Lin, linear; Quad, quadratic.

### 3.2 Effect of dietary fresh Amla fruit supplementation on rumen bacterial composition

**Figure 1:**
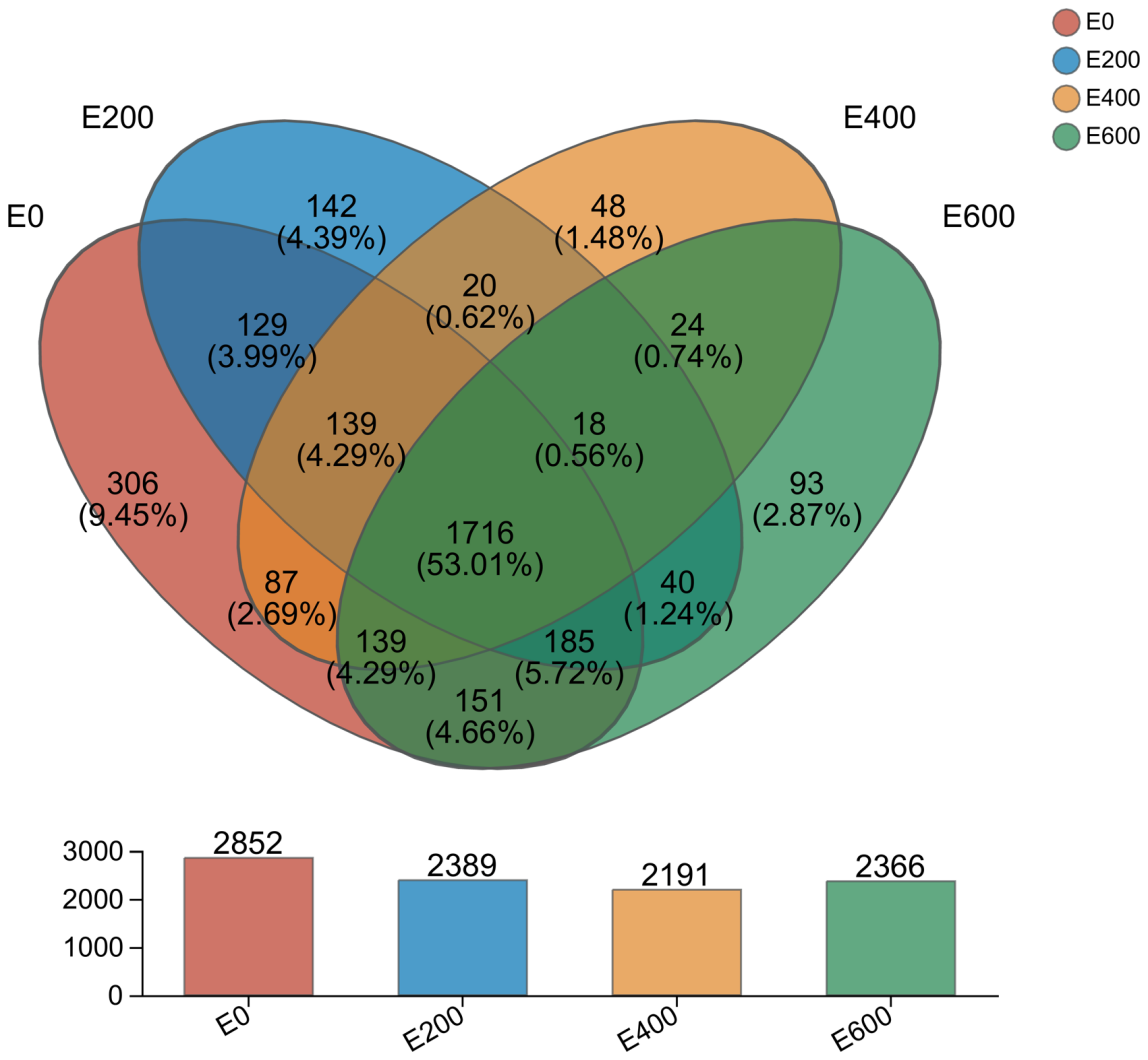
Shared and unique OTUs across different treatment groups.

In total, 7,448,697 sequences were optimized, each consisting of 3,075,340,663 bases and an average length of 412 bp. We analyzed the bacterial composition of rumen microbiota by sequencing the V3-V4 region of the 16S rRNA gene in the rumen samples. Taxonomic analysis revealed the detection of 28 phyla, 62 classes, 145 orders, 256 families, 550 genera, and 1,022 species of rumen bacteria across the four treatment groups. We clustered high-quality reads into 3,250 microbial OTUs that shared 97% similarity, of which 1,726 OTUs were present in all groups, amounting to 53.11% of the total OTUs. The control group (E0, 0 g/d) had the highest number of unique OTUs (317, 9.75%), followed by E200 (132, 4.06%) and E600 (87, 2.68%), and the lowest was in E400 FAF groups (46, 1.42%) (Figure 1).

### 3.3 Effect of dietary fresh Amla fruit supplementation on the relative abundance of bacteria

**Figure 2:**
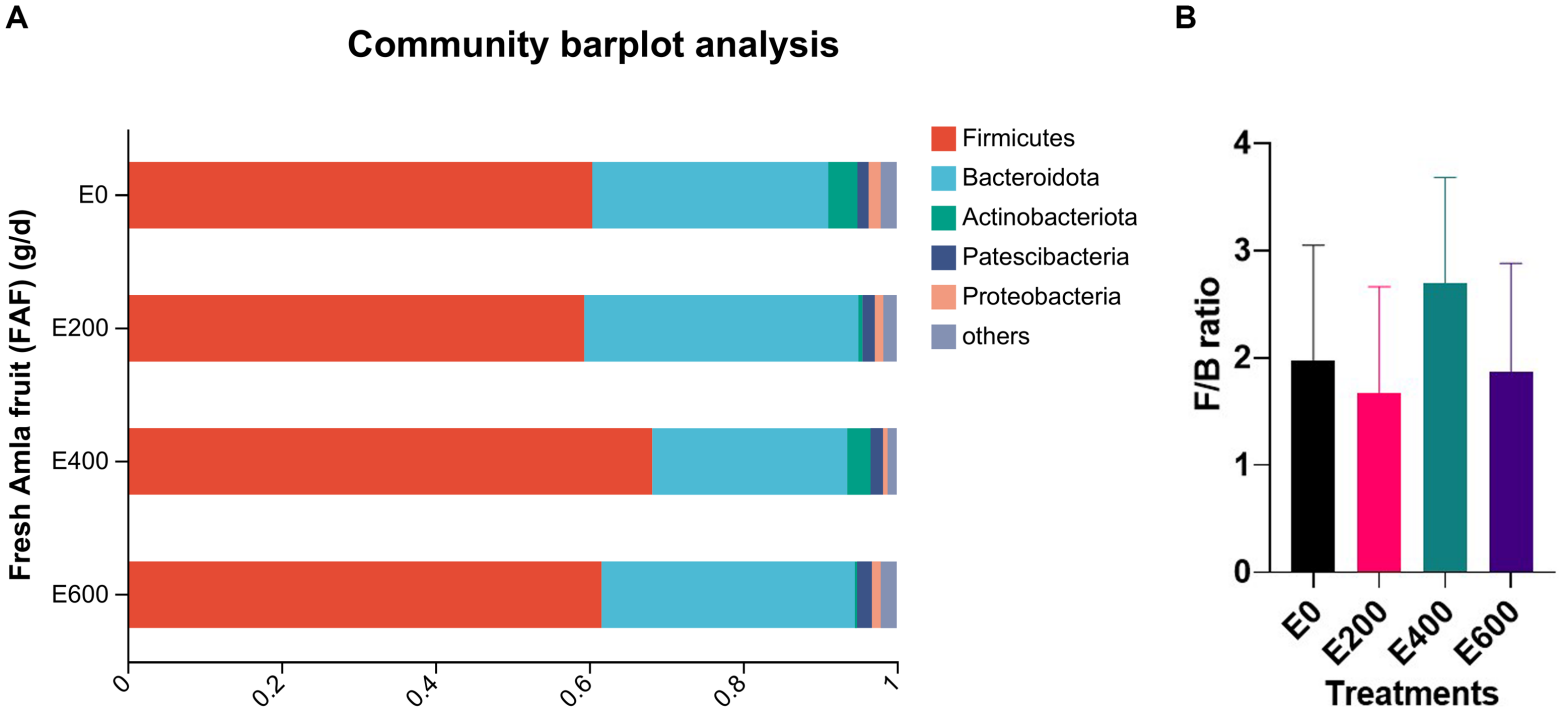
**(A)** Composition of microbiota community at the phylum level. Proportions represented the average relative abundance of microbes in different groups (Note E 0 = control; E 200 = 200 g/d FAF; E400 = 400 g/d FAF and E600 = 600 g/d FAF). **(B)** Firmicutes to Bacteriodetes ratio in different groups. Mention these are from cows (*n* =?) as above.

Amla fruit supplementation impacted the abundance of rumen bacteria. According to the taxonomic study, 28 bacterial phyla were identified. Among them, Firmicutes (61.8 ± 13.02%) and Bacteroidota (31.0 ± 12.48%) were the most prevalent, followed by Actinobacteriota (2.54 ± 8.96%), Patescibacteria (1.58 ± 1.12%), and Proteobacteria (1.25 ± 1.70%), as shown in Figure 2A and Supplementary Table S3. The dose of fresh Amla fruit (FAF) affected the abundance of the main phyla. The group of cows that supplemented 400 g/day FAF showed the highest abundance of Firmicutes (68.23 ± 9.26%) and the lowest abundance of Bacteroidota (25.33 ± 9.37%) compared with other groups. However, the group of cows that supplemented 200 g/day FAF had the highest abundance of Bacteroidota (35.63 ± 9.35%) among all the groups.

**Figure 3:**
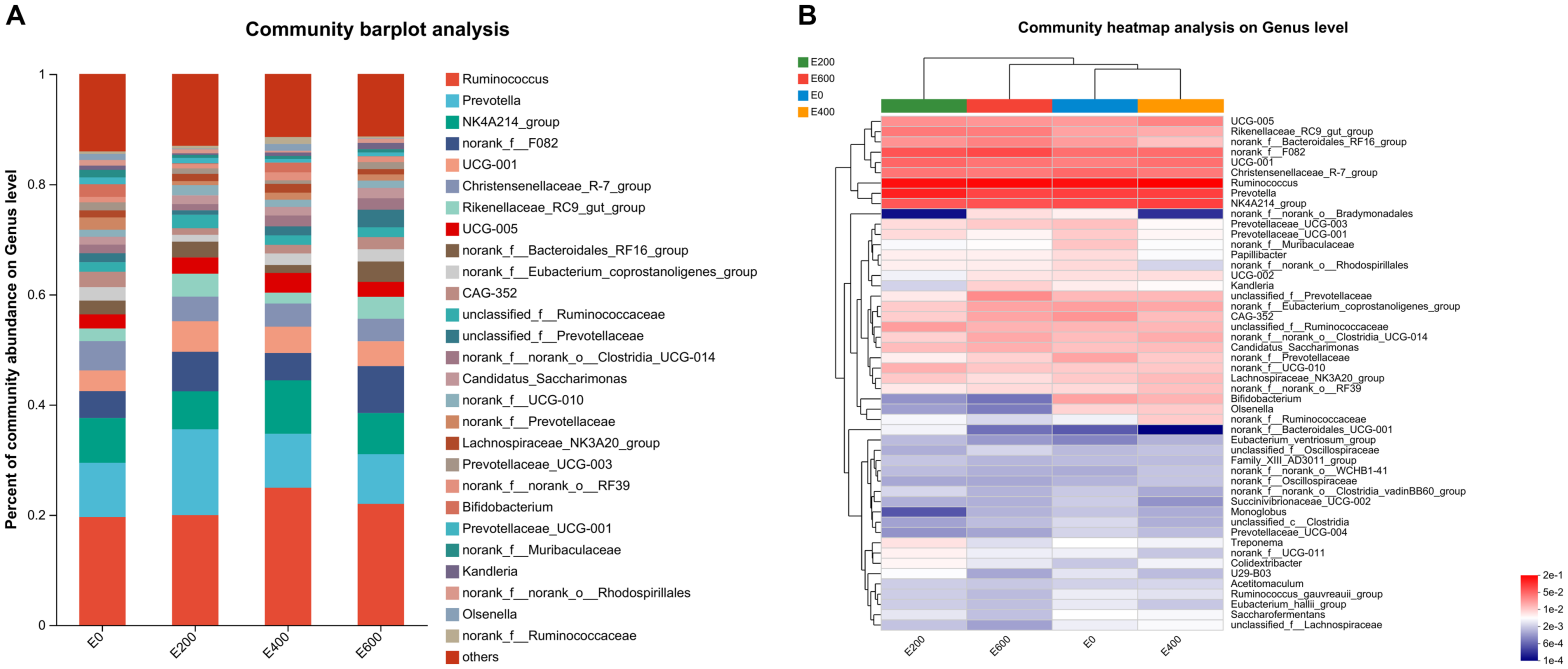
**(A)** Composition of microbiota community at the genus level. Proportions represented the average relative abundance of microbes in different community bar diagrams. **(B)** Microbial community heatmaps.

Figures 3A,B document the impact of fresh Amla fruit (FAF) on bacterial genera, with varying effects depending on the dosage administered. The study identified 29 bacterial taxa at the genus level, each with a relative abundance exceeding 0.10%. *Ruminococcus* was the most abundant, comprising 20.5 ± 11.1%, followed by *Prevotella* at 12.0 ± 9.85%, *Oscillospiraceae NK4A214_group* at 7.95 ± 3.41%, norank_f_F082 at 6.00 ± 5.54%, *Christensenellaceae R-7 group* at 4.70 ± 2.31%, *Prevotellaceae UCG-001* at 4.119 ± 6.36%, and *Rikenellaceae RC9 gut group* at 2.89 ± 2.87%. Additionally, *Oscillospiraceae UCG-005, norank_f Bacteroidales RF16 group*, and *Eubacterium coprostanoligenes group* were found to be abundant at 2.75 ± 2.28%, 2.50 ± 2.45%, and 2.07 ± 1.62%, respectively.

### 3.4 Effect of dietary fresh Amla fruit supplementation on taxonomic differences of rumen microbiota

**Figure 4:**
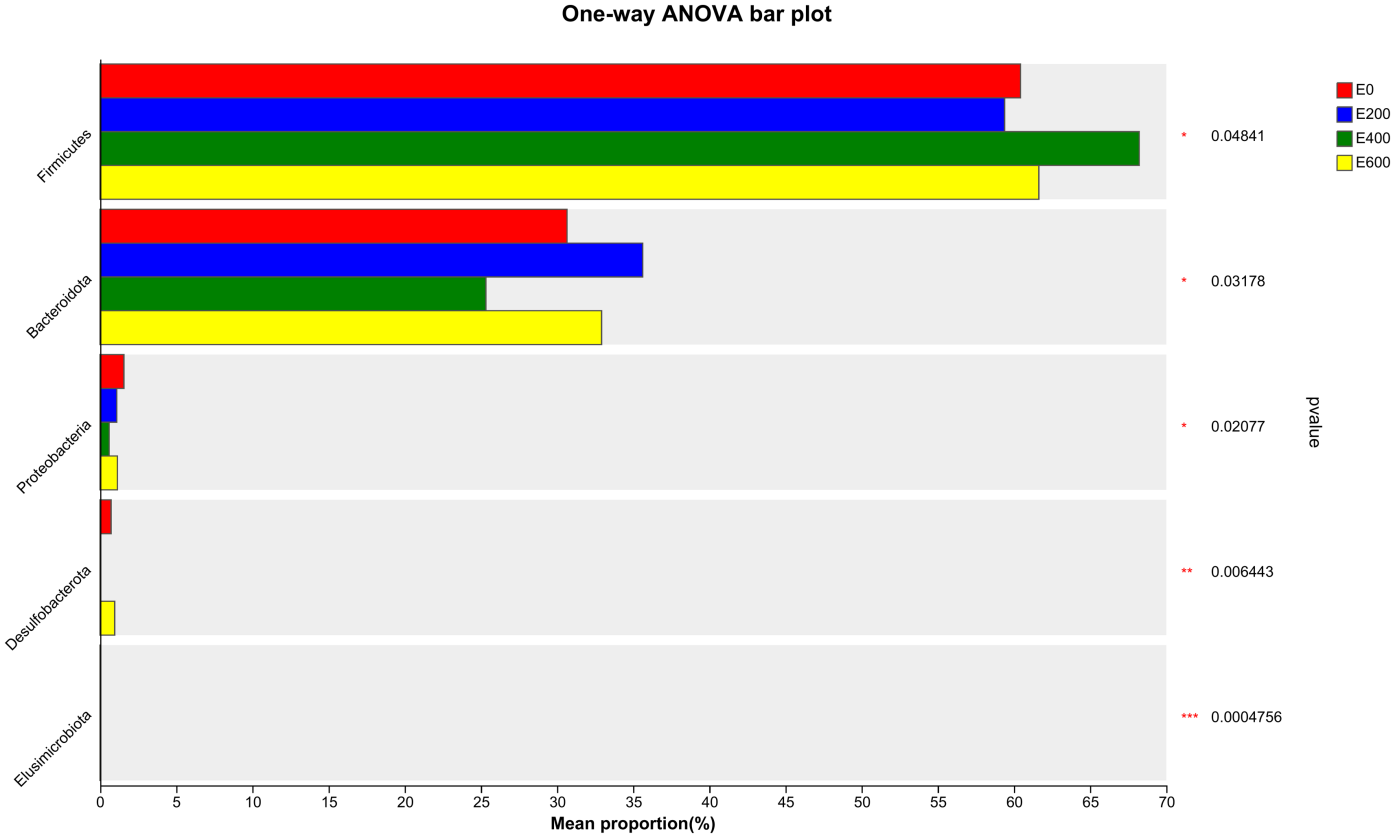
Microbiota community presented significantly different proportions (%) at the phylum level. Proportions represented the average relative abundance of microbes in different groups.

Fresh Amla fruit impacted the differences in bacterial species abundance among treatment groups and are shown in Figure 4 and Table 2. The results showed that administering 400 g/d of FAF resulted in an increased relative abundance of Firmicutes (*p* = 0.048), while Bacteroideta, Proteobacteria, and Elusimicrobiota decreased (< 0.032, 0.021, and < 0.001, respectively) compared with the other groups. Both 200 g/d and 400 g/d FAF supplementation led to a reduction in the relative abundance of Desulfobacterota compared with the control and 600 g/d FAF (*p* = 0.006) cows’ group.

**Table 2:** Effect of Amla fruit supplementation on the relative abundance of ruminal bacteria community at the phylum level.

PHYLUM	Fresh Amla fruit supplementation (g/d)				SD	*p*-value
	0	200	400	600		
Firmicutes	60.4^b^	59.4^b^	68.2^a^	61.6^ab^	13.02	0.048
Bacteroidota	30.7^b^	35.6^a^	25.3^b^	32.9^ab^	12.48	0.032
Actinobacteriota	3.79	0.53	3.05	0.29	8.96	0.078
Patescibacteria	1.46	1.60	1.60	1.92	1.12	0.406
Proteobacteria	1.57^a^	1.10^ab^	0.59^b^	1.14^a^	1.70	0.021
Spirochaetota	0.76	0.96	0.54	0.38	0.99	0.077
Desulfobacterota	0.72^a^	0.03^b^	0.04^b^	0.96^a^	1.41	0.006
Elusimicrobiota	0.04^a^	0.04^a^	0.01^b^	0.05^a^	0.04	<0.001

Arithmetic means in the same row within each dose with different letters differ significantly (*P* < 0.05).

At 200 g/d FAF, the relative abundance of Bacteroidota was higher than the control and 400 g/d FAF (*p* = 0.032). However, no difference in bacterial relative abundance was observed between the control and 600 g/d FAF supplementation at the phylum level. These findings suggest that FAF supplementation affects the bacterial phyla composition in dairy cows in a dose-dependent manner.

**Figure 5:**
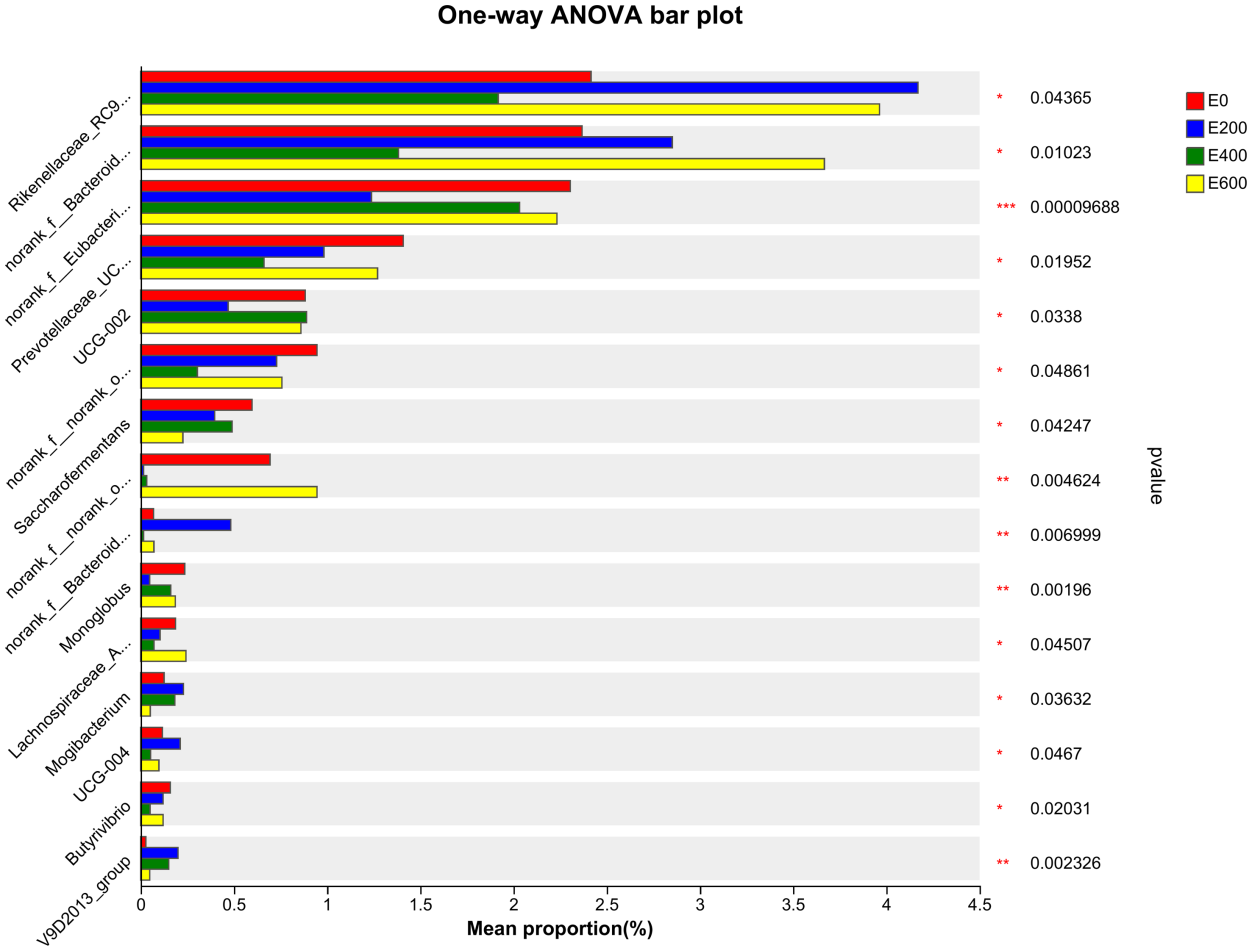
Microbiota community presented significantly different proportions (%) at the genus level. Proportions represented the average relative abundance of microbial in different groups.

The results presented in Figure 5 and Supplementary Table S4 indicate that cows fed different amount of fresh Amla fruit (FAF) had varying relative abundances of rumen bacteria at the genus level. Specifically, cows that consumed 200 g/d and 600 g/d of FAF had a higher relative abundance of *Rikenellaceae RC9 gut group*, *norank_f p-251-o5, and norank_o Izemoplasmatales* compared with the 400 g/d and control groups (*p* = 0.044, 0.011, and < 0.001, respectively). On the other hand, the 200 g/d and 600 g/d FAF cows had a lower relative abundance of *norank_o Absconditabacteriales SR1* (*p* = 0.014) than the different groups. Additionally, cows fed 600 g/d FAF had a higher relative abundance of the *Bacteroidales RF16* group than those provided 400 g/d FAF and the control group (*p* = 0.01). Moreover, cows that consumed 200 g/d FAF had a lower relative abundance of *Eubacterium coprostanoligenes group, Oscillospiraceae UCG-002, Monoglobus, Butyricicoccaceae UCG-009, Lysinibacillus,* and *Thermoactinomyces* than the other groups (*p* < 0.001, 0.034, 0.002, < 0.001, 0.002, and 0.002, respectively). Despite this, the 200 g/d FAF cows had higher levels of *Bacteroidales UCG-001, Prevotellaceae UCG-004*, and *Anaeroplasma* than other treatment groups (*p* = 0.007, 0.047, and 0.029, respectively).

**Figure 6:**
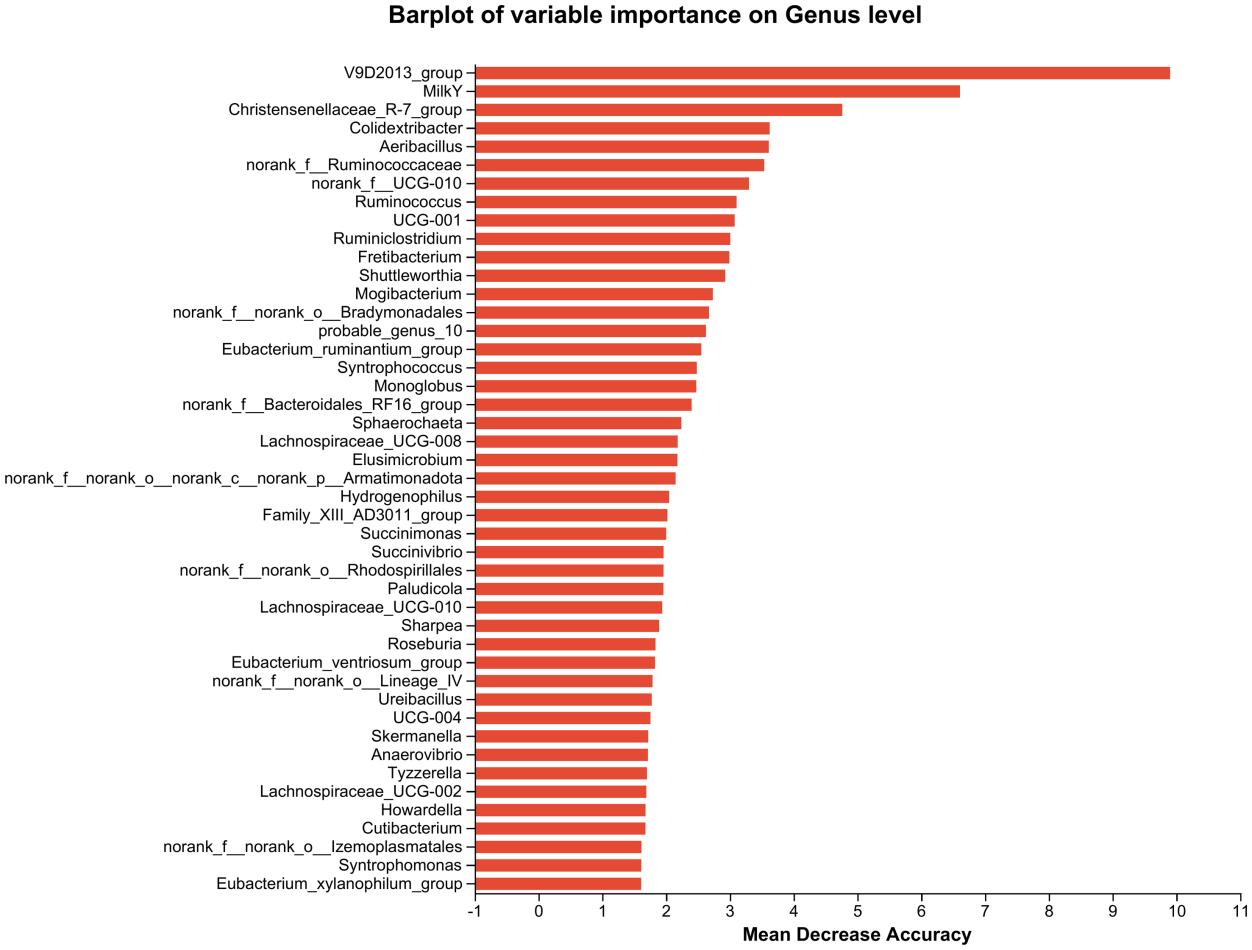
Random Forest species abundance statistics screened out the important species using mean decrease accuracy and then cross-validated each model (default: 10 times) and plotted the ROC curve (the species importance ranking diagram of iconic species obtained based on random forest reflects the ability of different species to influence the accuracy of the classification model. Legend: Species importance ranking chart, the Y-axis is the importance measure (such as species), the X-axis is equal to the importance measurement value/standard deviation value of the species; and the Y-axis corresponds to the species names sorted by importance.

Furthermore, cows that consumed 200 g/d and 400 g/d FAF had a lower relative abundance of *Prevotellaceae UCG-003, norank_o Bradymonadales, Lachnospiraceae AC2044 group, Succinimonas, and Pseudomonas* (*p* = 0.02, 0.007, 0.045, 0.006, and 0.002, respectively). In contrast, the 200 g/d and 400 g/d groups showed an increase in the relative abundance of the *Oscillospiraceae V9D2013 group* (*p* = 0.002) and an important biomarker for milk yield (Figure 6).

Additionally, supplementing with 400 g/d reduced the relative abundance of *norank_o Rhodospirillales UCG-004, Butyrivibrio, Elusimicrobium, Streptococcus, Endomicrobium*, and *Oscillibacter* (*p* = 0.007, 0.047, 0.020, 0.001, 0.038, 0.047, and 0.002, respectively). However, 400 g/d supplementation increased the levels of the *Prevotellaceae YAB2003 group* (*p* = 0.02) compared with the other cow groups. These findings demonstrate that the doses of FAF had a dose-dependent impact on bacterial genera in lactating cows.

**Figure 7:**
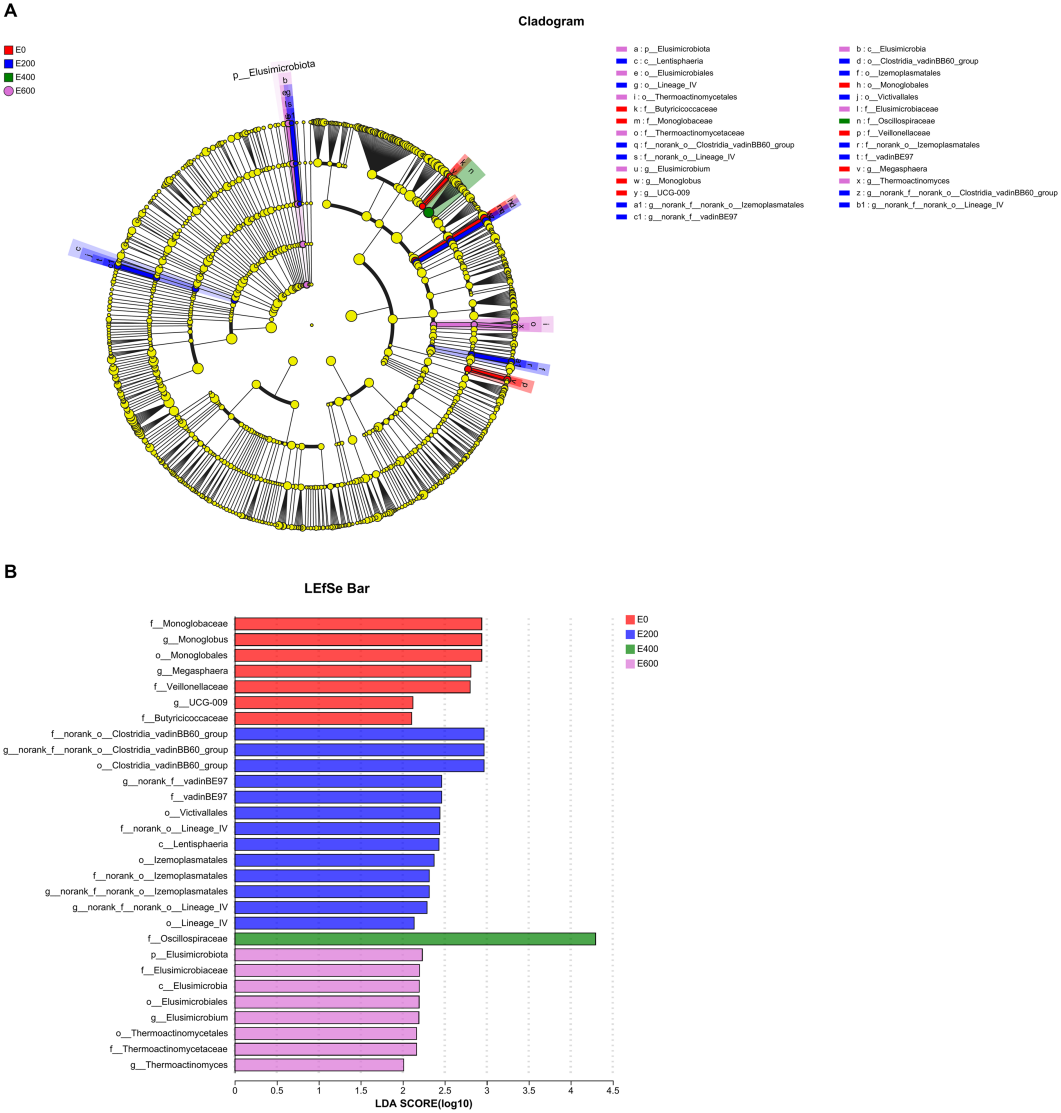
Differential abundant microbial taxa displayed by **(A)** cladogram and **(B)** barplot at the genus level affected by fresh Amla fruit supplementation at different groups detected using linear discriminant analysis (LDA) effect size (LEfSe) with an LDA score of ≥2.0.

Using linear discriminant analysis (LDA) effect size (Figures 7A,B), the impact of fresh Amla fruit (FAF) on bacterial communities found 29 bacterial biomarkers with LDA scores above 2.0 and *p* < 0.05 among the four groups. The Firmicutes phylum was the most prevalent in all groups, while the Elusimicrobiota phylum was only found in the 200 g/d FAF and 600 g/d FAF groups. The Verrucomicrobiota phylum was significantly abundant in the rumen of the 200 g/d FAF group compared with the other three groups (LDA > 2.0, *p* < 0.05). In the control group (0 g/d FAF), *Mongoglobaceae, Mongolobus, Monoglobales, Veillonellaceae, Megasphaera, UCG-009*, and *Butyricicoccaceae* were the most differentially abundant bacterial taxa. In the 200 g/d FAF group, the most differentially abundant bacterial taxa were *Clostridia_vadinBB60 group, vadinBE97, Victivallales, Lentisphaeria, lzemoplasmatales,* and *Lineage_IV*. The most differentially abundant bacterial taxa in the 400 g/d FAF group was *Oscillospiraceae*, while in the 600 g/d FAF group, *Elusimicrobium* and T*hermoactinomyces* were the most differentially abundant bacterial taxa.

### 3.5 Effect of dietary fresh Amla fruit supplementation on ruminal fermentation characteristics

In a companion manuscript, Tilahun et al. (2022a) have already documented the impacts of the effects of fresh Amla fruit (FAF) supplementation on ruminal fermentation characteristics. As FAF doses increased, the total concentration of volatile fatty acids (VFAs) increased quadratically but impacted the molar proportion of acetate and propionate cubically. Rumen NH_3_-N concentrations varied based on the FAF dosage, with the 200 g/d group having the lowest and the 600 g/d group having the highest concentration.

### 3.6 Correlations between bacterial genera and rumen fermentation parameters

**Figure 8:**
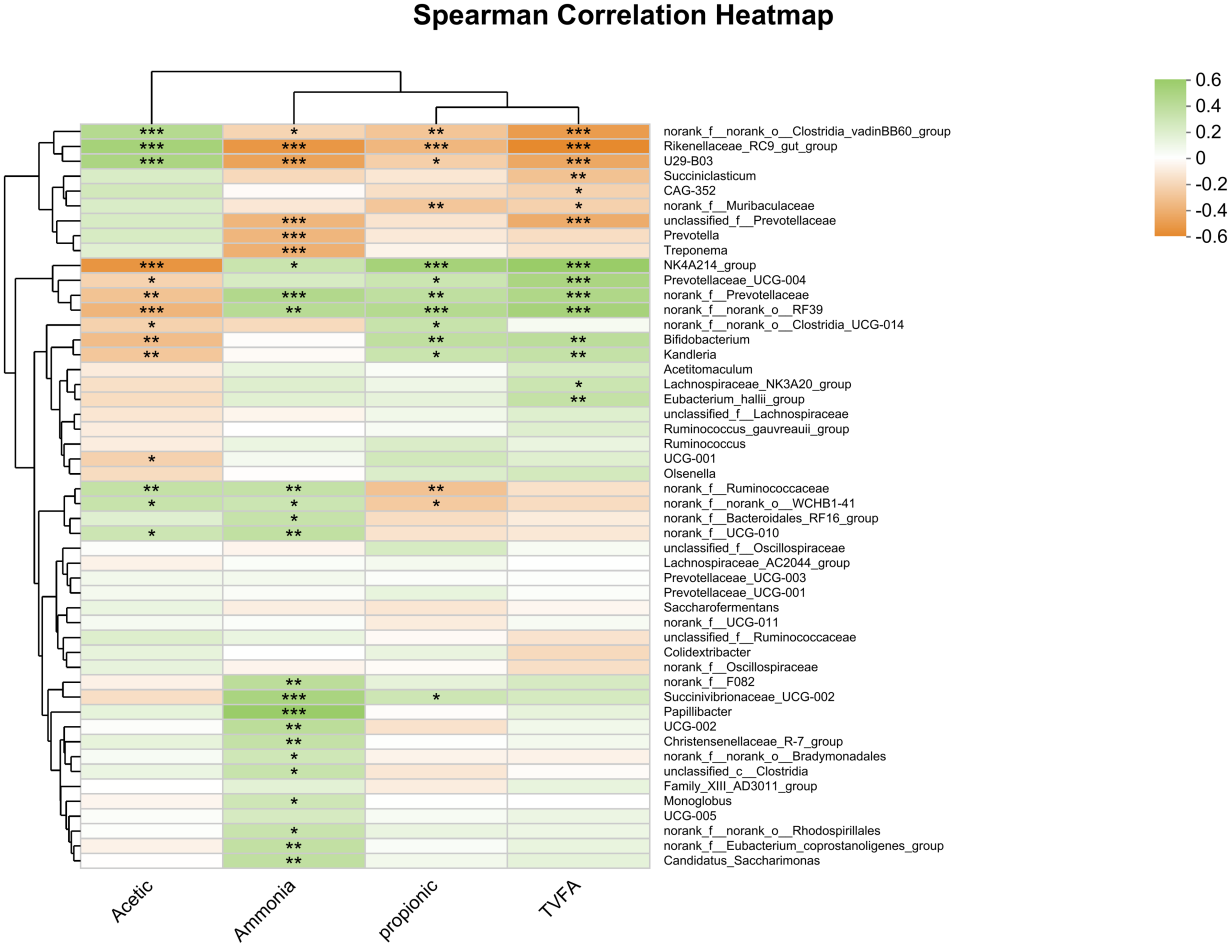
Heatmap of the correlation analysis between bacterial genera and rumen fermentation parameters. *0.01 < *p* ≤ 0.05, **0.001 < *p* ≤ 0.01, and ***p ≤ 0.001.

The findings indicate that *Oscillospiraceae, Bacilli RF39, norank_f Prevotellaceae,* and *Bifidobacterium* were positively correlated with the ruminal concentration of total VFA and the molar proportion of propionate (Figure 8). Conversely, the *Rikenellaceae RC9 gut group* and *Clostridia vadinBB60 group* were negatively correlated with both ruminal total VFA concentrations and the molar proportion of propionate. Additionally, the concentration of total VFA was negatively correlated with *Rikenellaceae U29-B03*, *unclassified f_Prevotellaceae*, and *Succiniclasticum*. Moreover, total VFA concentrations were positively correlated with *Prevotellaceae*, while the molar proportion of propionate was negatively correlated with *Ruminococcaceae*.

Additionally, the molar proportion of acetate was positively correlated with the relative abundance of the *Rikenellaceae RC9 gut group* (*r* = 0.450, *p* ≤ 0.0001), *Rikenellaceae U29-B03* (*r* = 0.417, *p* ≤ 0.001), and *Clostridia vadinBB60* (*r* = 0.367, *p* ≤ 0.001). Conversely, the molar proportion of acetate was negatively correlated with the relative abundance of *Oscillospiraceae NK4A214 group* (*r* = −0.525, *p* ≤ 0.0001), *norank_o__RF39* (*r* = −0.373, *p* ≤ 0.001), *Bifidobacterium* (*r* = −0.335, *p* = 0.002), and *norank f_ Prevotellaceae* (*r* = −0.311, *p* = 0.005). These findings provide valuable insights into the relationship between the molar proportion of acetate, propionate, and TVFA and the relative abundance of rumen bacteria is associated with specific taxa.

Furthermore, the concentration of ammonia nitrogen (NH_3_-N) was positively correlated with the relative abundance of *Papillibacter* (*r* = 0.510, *p* < 0.0001), *Succinivibrionaceae UCG-002* (*r* = 0.427, *p* < 0.0001), *norank_f Prevotellaceae* (*r* = 0.382, *p* < 0.001), *norank_o__RF39* (*r* = 0.349, *p* = 0.001), *Bacteroidales norank_f__F082* (*r* = 0.331, *p* = 0.003), *Oscillospiraceae UCG-002* (*r* = 0.331, *p* = 0.003), *Candidatus Saccharimonas* (*r* = 0.307, *p* = 0.005), and *Oscillospirales f__UCG-010* (*r* = 0.302, *p* = 0.006). Conversely, there was a negative correlation between the concentration of ammonia nitrogen (NH_3_-N) and the relative abundance of *Rikenellaceae RC9 gut group* (*r* = −0.516, *p* < 0.0001), *Rikenellaceae U29-B03* (*r* = −0.459, *p* < 0.0001), *Treponema* (*r* = −0.396, *p* < 0.001), *unclassified_f__Prevotellaceae* (*r* = −0.371, *p* < 0.001), and *Prevotella* (*r* = −0.369, *p* < 0.001).

## 4 Discussion

According to our recent study, supplementation of 400 g/d fresh Amla fruit (FAF) to cows reduced ruminal protozoa counts and increased milk protein and nitrogen efficiency without any negative impact on milk production (Tilahun et al., 2022a). However, the same study found that a higher dosage of 600 g/d of FAF led to a decline in milk yield and an increase in NH_3_-N concentrations, as compared with 200 or 400 g/d of FAF supplementation. Moreover, Tilahun et al. (2022b) reported that supplementing cows with fresh Amla fruit at a dosage of 200 or 400 g/day can increase the concentration of healthful fatty acids in milk and modulate biohydrogenation. Moreover, this current study also shows a shift in microbial compositions based on different dosage levels. These results signify the promising impact of FAF as a new supplemental feed in positively influencing milk quality, animal health, and nitrogen efficiency.

The present study examines the effects of fresh *Phyllanthus emblica* (Amla) fruit, which is rich in hydrolyzable tannins, phenolic acids, flavonoids, and other compounds (Tilahun et al., 2022b), on rumen microbial composition, as well as its correlation with rumen fermentation. Rumen microbial populations are critical for their digestive processes, nutrient intake, and overall health (Gao et al., 2022; Wang G. et al., 2023). Our research revealed that the core microbiota in the rumen is dominated by three phyla, namely, Firmicutes, Bacteroidota, and Actinobacteriota, which make up approximately 95.24% of the total microbial population (Figure 2A). Herein, we fed dairy cows a 50:50 ratio of concentrate and forage, with corn silage being the primary source of forage. The most dominant phylum in the rumen in our study was Firmicutes, followed by Bacteroidetes. This is consistent with the study by Zhang et al. (2021), which found Firmicutes to be the most prevalent phylum in moderate- and high-grain diets and silage-based forage diets. Additionally, Sun et al. (2019) reported that feeding PSM-rich Perilla leaf increased the number of phylum Firmicutes in cows, which is similar the findings of our study. The dominance of these two phyla and Actinobacteriota is crucial for the breakdown of feeds, nutrient supply, dry matter intake, and animal health. Various studies, including (Min et al., 2015; Diaz Carrasco et al., 2017; Sun et al., 2019), have also highlighted the importance of Firmicutes and Bacteroidetes in the rumen microbiome.

Cows that received 400 g/day of FAF supplementation showed a significant increase in the abundance of Firmicutes and a decrease in the abundance of Bacteroidota and Proteobacteria. This resulted in a high ratio of Firmicutes to Bacteroides, as presented in Figure 2B, when compared with cows that received other doses. The increase in Firmicutes to Bacteroidetes ratio has been previously associated with the energy-harvesting capacity of the gut microbiota and milk-fat yield in dairy cattle (Ebeid et al., 2020). Furthermore, the abundance of Firmicutes in the rumen has been positively correlated with the average daily body weight gain in steers, suggesting that these bacteria play a crucial role in the feed efficiency of bovines (Diaz Carrasco et al., 2017). An increase in the Firmicutes to Bacteroidetes ratio has also been linked to improved intestinal health (Jang et al., 2020). Additionally, cows with higher milk yields were reported to have a greater abundance of Firmicutes and a lower abundance of Bacteroidetes and Proteobacteria than those with lower milk yields (Tong et al., 2018). Therefore, the relatively high abundance of Firmicutes and the high ratio of Firmicutes to Bacteroidetes at 400 g/day FAF supplementation is one of the reasons why our accompanying study reported enhanced milk performance, milk nitrogen efficiency, milk fatty acid profiles, and antioxidant capacity with up to 400 g/day fresh Amla fruit supplementation (Tilahun et al., 2022a,b).

Moreover, compared to the other groups, the addition of 400 g/d of FAF resulted in a decrease in the relative abundance of Proteobacteria and Elusimicrobiota, as indicated in Table 2. Similarly, De Nardi et al. (2016) reported that supplementing heifers with a mixture of phenolic compounds (flavonoids) reduced the phyla Proteobacteria. Therefore, the phenolics, tannins, and flavonoids in fresh Amla fruit reduced the relative abundance of phyla Proteobacteria at 400 g/d doses dependently compared with other groups of cows. The Proteobacteria group of bacteria includes several opportunistic pathogens sensitive to dietary change, such as *Escherichia*, *Campylobacter*, *Vibrio*, *Helicobacter*, and *Salmonella* (Sezenna, 2011; Bäumler and Sperandio, 2016). A greater prevalence of Proteobacteria is often associated with antimicrobial resistance and pathogenicity, indicating dysbiosis—an imbalance in microbial populations (Durso et al., 2012; Auffret et al., 2017). This dysbiosis is prevalent in animals that fed a high-concentrate diet, leading to subacute ruminal acidosis (SARA). Additionally, studies conducted by Rizzatti et al. (2017) have shown that Proteobacteria is a common factor in human diseases.

Several studies have shown that the relative abundance of Proteobacteria in dairy heifers increases linearly with higher dietary concentrate levels (high energy density), particularly in those feds with corn silage-based diets and high concentrate diets (Auffret et al., 2017; Deusch et al., 2017; Zhang et al., 2018). Moreover, the more efficient steers experienced a significant decrease in the abundance of Proteobacteria in their hindgut during the feedlot phase, and the genus *Proteobacteria* was positively correlated with high residual feed intake (Auffret et al., 2020; Lourenco et al., 2022). Our study indicated that adding FAF at 400 g/d can lower Proteobacteria levels, potentially reducing pathogens that cause inflammation and microbial dysbiosis, leading to SARA. These findings suggest that supplementing FAF at 400 g/d might be a helpful way to reduce the impact of SARA caused by high-grain diets, ultimately improving animal efficiency and rumen health.

Desulfobacterota, previously known as Deltaproteobacteria, are known for its ability to reduce sulfate, break down aromatic hydrocarbons, and fix nitrogen (David et al., 2014), and were decreased in dairy cows that fed 200 and 400 g/d of FAF supplements. Desulfobacterota also contribute to butyrate degradation by carrying out a butyrate beta-oxidation pathway (Janssen and Schink, 1995), and it has been suggested that the Desulfobacterota phylum could potentially release lipopolysaccharide (LPS), which may trigger inflammatory injuries or worsen energy metabolism abnormalities (Huang et al., 2021). In another study, Zou et al. (2022) found that heat stress increased the relative abundance of the Desulfobacterota phylum in beef cattle, while nicotinic acid (niacin) supplementation decreased in the relative abundance. Our result showed reduction of Desulfobacterota phylum when the dairy cows are supplemented with 200 and 400 g/d of FAF doses, indicating that these doses of FAF may create a healthier environment in the rumen and hindgut of the cows by minimizing the release of lipopolysaccharide (LPS) (Huang et al., 2021). Additionally, supplementing FAF in the feed may also mitigate heat stress conditions in dairy cows (Zou et al., 2022). However, further research is required to validate this potential benefit. At the genus level, the *Oscillospiraceae V9D2013 group* increased in cattle fed 200 and 400 g/d FAF supplementation. Chen J. et al. (2022) report that chlorogenic acid treatment increased the abundance of butyrate-producing bacteria and the V9D2013 group, leading to improved weight gain in rabbits. However, Jing et al. (2022) found negative correlations between the *V9D2013 group* and terminal weight, net gain, and daily gain of rabbits. These inconsistent results may be due to differences in feed type and environmental conditions. Interestingly, as shown in Figure 6, random forest species abundance statistics of our study identified *V9D2013* as an essential biomarker related to milk yield. Our accompany studies (Tilahun et al., 2022a,b) also recommended 200 and 400 g/d FAF dose levels due to their positive influence on rumen fermentation, nitrogen efficiency, milk yield, and desirable fatty acids. The higher abundance of *V9D2013* in these groups may explain the strong correlation between *V9D2013* and milk yield, as it has the potential to produce higher butyrate, according to various studies. For instance, Seymour et al. (2005)) found that the relationship between milk yield and rumen VFA was least for acetate and greatest for butyrate. Additionally, butyrate producers are essential for maintaining gut health by inhibiting oxidative stress (Guilloteau et al., 2010; Hu et al., 2013). However, further research is necessary to validate the use of *V9D2013* as a biomarker for milk yield and consider it a potential probiotic bacterium.

The relative abundance of the family Oscillospiraceae increased significantly at 400 g/d FAF feeding dose, and it was the only potential biomarker identified for this treatment group (Figures 7A,B). Liu et al. (2023) discovered that different tannin sources added to broiler diets had varying effects on Oscillospiraceae, with enrichment observed only in *Castanea sativa* tannin and *Schinopsis lorentzii* tannin; these specific tannins degrade various structural carbohydrates and produce butyrate, positively impacting broilers’ liver protection. Furthermore, *Aurantii Fructus immaturus* flavonoid extract increased the proportion of Oscillospiraceae and ameliorated colitis induced by dextran sulfate sodium in mice (Chen S-.Y. et al., 2022). Molino et al. (2022) also reported an increase in Oscillospiraceae with tannin supplementation and identified them as markers of a healthy gut due to their role in protein degradation and mucin breakdown. Interestingly, some studies have shown that Oscillospiraceae is enriched in the microbiome of human athletes related to high performance (Han et al., 2020), indicating that dairy cows may also benefit from increased energy efficiency to improve their performance.

Moreover, other studies have shown that Oscillospiraceae is abundant in healthy small ruminants and calves (Fan et al., 2021; Wang X. et al., 2023). Aqueous extract of *Tetrastigma hemsleyanum* leaves in mice increased the abundance of Oscillospiraceae and reduced intestinal inflammation (Wang J. et al., 2023). Free-range ruminants have also been found to have an increased proportion of Oscillospiraceae, which has the potential to break down benzoic acid and has a positive correlation with resistance to parasitic infections (Fu et al., 2022). Oscillospiraceae families are considered to be potential next-generation probiotics (Van den Abbeele et al., 2022) and might also be considered as a biomarker for successful pregnancy (Koester et al., 2021) because of its role as a butyrate producer. Butyrate aids the host in forming tight cell junctions in the epithelial layer, which can potentially prevent bacterial infection that could ultimately lead to spontaneous abortion (Peng et al., 2009; Koester et al., 2021). The evidence presented above indicates that Oscillospiraceae, a type of bacterium, were enhanced in dairy cows that were fed a 400 g/d FAF-supplemented diet. This suggests that Amla fruit has the potential to improve the production of butyrate-producing bacteria, which is highly correlated with milk production, rumen homeostasis, and overall health. Our accompanying study (Tilahun et al., 2022b) supported this, which showed high antioxidant capacity in milk and other desirable performance for this group of dairy cows. It encourages further investigation using Amla fruit in the future.

Furthermore, *Oscillospiraceae NK4A214 group* has a positive correlation with the concentration of total VFA and the molar proportion of propionate in ruminal fermentation from the degradation of complex carbohydrates found in plants (Konikoff and Gophna, 2016) and is thought to play a role in fiber degradation in the rumen due to its abundant production of endo-1, 4-beta-xylanase, and cellulase genes (Yi et al., 2022). A study by Wang G. et al. (2023) reported that the *Oscillospiraceae NK4A214 group* produces propionate and butyrate in goats, which is consistent with our results. However, another study by Guo et al. (2023) found a positive correlation between the group comprising *NK4A214* and the concentration of acetic acid in calves, which could be due to variations in feed and species and underscores the need for further research in this area.

The present research indicates a significant positive correlation between the molar proportion of acetate and the relative abundance of the *Rikenellaceae RC9 gut group*, with negative correlations observed for propionate and total VFA. This finding is consistent with previous studies, which reported a negative correlation between the *RC9 gut group* and propionate (Li et al., 2022; Zhang et al., 2023). The negative correlation could be attributed to the production of acetate by some members of the *RC9 gut group*, as reported by Su et al. (2014). The *RC9 gut group* is crucial for fiber digestion, rumen fermentation pattern, and epithelial development (Qiu et al., 2022). However, other studies have indicated that it may have a negative impact on the enhancement of ruminal N utilization efficiency (Zhang et al., 2023). Our study revealed that the 400 g/d FAF supplementation group had a significantly lower abundance of the *Rikenellaceae RC9 gut group* than the other groups, underscoring the importance of this dose in improving nitrogen utilization efficiency. As previously reported, this group also showed higher nitrogen efficiency (Tilahun et al., 2022a).

Harrison et al. (2018) described the *Clostridiales vadinBB60 group* as a microbe that produces short-chain fatty acids (SCFAs). Our research shows a positive correlation between *vadinBB60* and propionate and rumen TVFA proportions. This aligns with the findings by Zhang et al. (2018), suggesting that *vadinBB60* is connected to propionate production. Moreover, Tilahun et al. (2022a) demonstrated that propionate concentration was higher at 200 g/d FAF supplementations. Our research supports the use of the *Clostridiales vadinBB60 group* as a potential biomarker (score ~ 3.0) in LDA analysis for 200 g/d FAF supplementation (Figure 7B). Moreover, Fonseca et al. (2023) observed that animals with low residual feed intake (RFI) had a greater abundance of *vadinBB60* bacteria, supporting our present results that improved performance observed with 200 g/d FAF might be attributed to *vadinbB60* bacteria.

Furthermore, the abundance of the fiber-degrading bacteria *Oscillibacter* was significantly reduced in cows that were given 400 g/d of FAF supplements (Thoetkiattikul et al., 2013). Probiotic treatments can also reduce *Oscillibacter*, which is related to obesity-related metabolic disorders and is potentially harmful due to its pro-inflammatory effects (Kong et al., 2019). Patients suffering from stroke show an elevated amount of *Oscillibacter*, associated with gut permeability, deterioration in renal function, and host inflammation by altering the pyruvate metabolism pathway weighted toward anaerobic glycolysis (Kim et al., 2020). This finding is significant and underscores the potential of Amla fruit as a valuable dietary supplement for improving animal health.

Previous research by Nagaraja and Titgemeyer (2007) has shown that the genus *Succinimonas* is more abundant in the high-grain group and that this genus, especially *Succinimonas amylolytica*, is linked to subacute ruminal acidosis (SARA) when present in higher relative abundances (Li et al., 2014). As reported by Grazziotin et al. (2020), the introduction of phytochemicals such as tannins and *Capsicum* species to the diets of lactating dairy cows can reduce the presence of *Succinimonas* bacteria, which is correlated with lower feed efficiency in ruminants. By decreasing the abundance of *Succinimonas* and *Streptococcus*, milk production and efficiency can be increased through higher levels of volatile fatty acids (Grazziotin et al., 2020). Our accompanying study by Tilahun et al. (2022a) indicates that FAF supplementation at 200 and 400 g/day results in an increase in milk production, which may be a factor for improvement.

Furthermore, including 400 g/day of FAF in the diet has effectively reduced the relative abundance of *Butyrivibrio* bacteria present in the rumen. This is instrumental in limiting biohydrogenation and increasing the proportion of unsaturated fatty acid, which is desirable in milk and beneficial to human health (Adler et al., 2013). Tilahun et al. (2022b) showed a significant improvement in desirable fatty acid profile of milk when FAF was included in the diet at a daily dose of 400 grams. Authors reported a remarkable increase in C22:6 (docosahexaenoic acid, DHA) and C20:5 (eicosapentaenoic acid, EPA), both of which are nutritionally rich omega-3 fatty acids, and a decrease in undesirable milk fat proportion of C12:0, C14:0, and C16:0 at 400 g/day of FAF. Similarly, Dai et al. (2017) reported that *Butyrivibrio* had a linear negative correlation with C18:2 n-6 and C18:3 n-3 concentrations. Moreover, Vasta et al. (2010) said that phenolic compounds suppress the last step of biohydrogenation of polyunsaturated fatty acid (PUFA), which was achieved by inhibiting the proliferation and activity of *Butyrivibrio proteoclasticus*. Moreover, *Butyrivibrio* spp. was suggested to isomerize 18:2n-6 to cis-9,trans-11 CLA more rapidly than other bacterial species (Dewanckele et al., 2020). The above studies suggest that the phytochemicals present in fresh Amla fruit can inhibit *Butyrivibrio* bacteria from interfering with biohydrogenation and enhance the production of desirable fatty acids in milk in a dose-dependent manner.

## 5 Conclusion

The aim of this investigation was to evaluate the feasibility of incorporating fresh Amla fruit (FAF) as a supplementary feed for livestock. The primary focus of the study was to investigate the impact of FAF on rumen microbes and the associated correlation with rumen fermentation. The study revealed several noteworthy findings concerning the utilization of FAF as a feed additive. It was observed that FAF had a significant effect on the composition of rumen microbes, where Firmicutes and Bacteroidota were the dominant phyla. Supplementation of FAF at 200 and 400 g/d levels reduced the presence of *Proteobacteria* and *Desulfobacteriota*, indicating enhanced efficiency and improved rumen health. The *V9D2013* group was identified as a biomarker for milk yield, and its relative abundance was higher at these supplementation levels.

Additionally, *Oscillospiraceae*, which have potential probiotic properties, showed a considerable increase at a supplementation level of 400 g/d FAF. In contrast, harmful microbes such as *Succinimonas* and *Streptococcus* decreased, while the lower count of *Butyrivibrio* bacteria explained the production of beneficial milk fatty acid. Overall, incorporating FAF improved nitrogen efficiency, desirable milk fatty acids, and antioxidant capacity. However, future research is needed to explore probiotic potential of FAF and its role in mitigating subacute ruminal acidosis while promoting animal health.

## Data availability statement

The datasets presented in this study can be found in online repositories. The names of the repository/repositories and accession number(s) can be found in the article/Supplementary material.

## Ethics statement

The animal studies were approved by the Institute of Animal Science (IAS), Chinese Academy of Agricultural Sciences (CAAS) guidelines (No. IAS20180115). The studies were conducted in accordance with the local legislation and institutional requirements. Written informed consent was obtained from the owners for the participation of their animals in this study.

## Author contributions

MT: Conceptualization, Data curation, Formal analysis, Methodology, Validation, Writing – original draft, Writing – review & editing. LM: Funding acquisition, Methodology, Project administration, Resources, Supervision, Validation, Writing – review & editing. TC: Supervision, Validation, Writing – review & editing. JX: Conceptualization, Funding acquisition, Investigation, Resources, Writing – review & editing. DB: Conceptualization, Funding acquisition, Investigation, Supervision, Validation, Visualization, Writing – review & editing.
