# Supplementary material for: The effect of Phyllanthus emblica (Amla) fruit supplementation on the rumen microbiota and its correlation with rumen fermentation in dairy cows
Source: Front Microbiol. 2024 May 13;15:1365681. doi: 10.3389/fmicb.2024.1365681 (PMC11128671; doi:10.3389/fmicb.2024.1365681)
Supplement: Supplementary file 1 [file Table_1.docx]

**Supplementary Table 1**. Schematic of experimental design and supplementation of fresh Amla fruit doses

| Cow | Weeks | | | | | | | |
| --- | --- | --- | --- | --- | --- | --- | --- | --- |
|  | Adaptation | Period 1 | | | Washout | Period 2 | | |
|  | -2 | 2 | 4 | 6 | 8 | 10 | 12 | 14 |
|  | Fresh Amla fruit (g/d) | | | | | | | |
| MY010 | 0 | 200 | 400 | 600 | 0 | 0 | 0 | 0 |
| MY001 | 0 | 200 | 400 | 600 | 0 | 0 | 0 | 0 |
| P71 | 0 | 200 | 400 | 600 | 0 | 0 | 0 | 0 |
| 090922 | 0 | 200 | 400 | 600 | 0 | 0 | 0 | 0 |
| 1108 | 0 | 0 | 0 | 0 | 0 | 200 | 400 | 600 |
| MY002 | 0 | 0 | 0 | 0 | 0 | 200 | 400 | 600 |
| P34 | 0 | 0 | 0 | 0 | 0 | 200 | 400 | 600 |
| P72 | 0 | 0 | 0 | 0 | 0 | 200 | 400 | 600 |
