# Supplementary material for: The effect of Phyllanthus emblica (Amla) fruit supplementation on the rumen microbiota and its correlation with rumen fermentation in dairy cows
Source: Front Microbiol. 2024 May 13;15:1365681. doi: 10.3389/fmicb.2024.1365681 (PMC11128671; doi:10.3389/fmicb.2024.1365681)
Supplement: Supplementary file 2 [file Table_2.docx]

**Supplementary Table 2**. Chemical composition of individual total mixed ration (TMR) ingredients and additive fresh Amla fruit (Least squares mean ± SE^*^)

| Item | Alfalfa hay | Corn silage | Concentrate mixture | Amla fruit | SE |
| --- | --- | --- | --- | --- | --- |
| Chemical composition (g/kg DM unless noted) | |  |  |  |  |
| Dry matter (DM) | 872 | 286 | 860 | 231 | 4.1 |
| Organic matter (OM) | 897 | 936 | 917 | 972 | 4.6 |
| Crude protein (CP) | 169 | 73.0 | 224 | 35.4 | 1.7 |
| Neutral detergent fiber (aNDF) | 387 | 413 | 172 | 347 | 5.2 |
| Acid detergent fiber (ADF) | 275 | 257 | 83.6 | 237 | 5.4 |
| Ether extract (EE) | 8.13 | 7.07 | 30.4 | 6.44 | 1.4 |
| Non-fiber carbohydrates (NFC)^1^ | 332 | 453 | 514 | 588 | 8.8 |
| Total phenolic content (mgTA/g^2^ DM) | - | - | - | 51.2 | 1.8 |
| Total flavonoid content (mgQT/g^3^ DM) | - | - | - | 87.8 | 1.6 |

^*^ , standard error; ^1^Non-fibre carbohydrates-Calculated as 100 − (NDF + CP + EE + ash); ^2^ mgTA/g; milligram tannic acid/gram ; ^3^ mgQT/g; milligram quercetin/gram
