# Supplementary material for: The effect of Phyllanthus emblica (Amla) fruit supplementation on the rumen microbiota and its correlation with rumen fermentation in dairy cows
Source: Front Microbiol. 2024 May 13;15:1365681. doi: 10.3389/fmicb.2024.1365681 (PMC11128671; doi:10.3389/fmicb.2024.1365681)
Supplement: Supplementary file 3 [file Table_3.docx]

**Supplementary Table 3:** Mean average relative abundance and statistical difference of the phyla in different groups.

| Species name | E0-Mean (%) | E0-Sd (%) | E200-Mean (%) | E200-Sd (%) | E400-Mean (%) | E400-Sd (%) | E600-Mean (%) | E600-Sd (%) | P-value |
| --- | --- | --- | --- | --- | --- | --- | --- | --- | --- |
| p__Firmicutes | 60.440 | 14.600 | 59.390 | 9.309 | 68.230 | 9.260 | 61.640 | 13.330 | 0.048 |
| p__Bacteroidota | 30.660 | 13.540 | 35.630 | 9.352 | 25.330 | 9.366 | 32.930 | 13.230 | 0.032 |
| p__Actinobacteriota | 3.785 | 11.610 | 0.526 | 0.578 | 3.049 | 8.435 | 0.290 | 0.386 | 0.078 |
| p__Patescibacteria | 1.463 | 1.068 | 1.599 | 0.993 | 1.595 | 1.588 | 1.923 | 0.846 | 0.406 |
| p__Proteobacteria | 1.565 | 2.239 | 1.098 | 1.022 | 0.594 | 0.574 | 1.136 | 0.701 | 0.021 |
| p__Spirochaetota | 0.764 | 1.087 | 0.959 | 1.192 | 0.542 | 0.912 | 0.381 | 0.298 | 0.077 |
| p__Desulfobacterota | 0.723 | 1.384 | 0.035 | 0.026 | 0.043 | 0.037 | 0.959 | 2.394 | 0.006 |
| p__Verrucomicrobiota | 0.199 | 0.205 | 0.283 | 0.217 | 0.255 | 0.408 | 0.203 | 0.162 | 0.580 |
| p__Cyanobacteria | 0.119 | 0.162 | 0.116 | 0.067 | 0.110 | 0.194 | 0.225 | 0.214 | 0.300 |
| p__Fibrobacterota | 0.073 | 0.133 | 0.182 | 0.402 | 0.041 | 0.039 | 0.038 | 0.028 | 0.210 |
| p__Synergistota | 0.086 | 0.104 | 0.053 | 0.039 | 0.064 | 0.044 | 0.079 | 0.062 | 0.247 |
| p__Armatimonadota | 0.023 | 0.056 | 0.013 | 0.011 | 0.095 | 0.249 | 0.048 | 0.080 | 0.161 |
| p__unclassified_k__norank_d__Bacteria | 0.031 | 0.036 | 0.039 | 0.041 | 0.019 | 0.040 | 0.084 | 0.169 | 0.349 |
| p__Elusimicrobiota | 0.045 | 0.051 | 0.044 | 0.037 | 0.014 | 0.018 | 0.050 | 0.037 | 0.000 |
| p__Chloroflexi | 0.024 | 0.047 | 0.024 | 0.025 | 0.016 | 0.035 | 0.015 | 0.036 | 0.796 |
| p__Fusobacteriota | 0.000 | 0.001 | 0.004 | 0.009 | 0.000 | 0.000 | 0.002 | 0.007 | NA |
| p__Acidobacteriota | 0.001 | 0.005 | 0.001 | 0.003 | 0.001 | 0.003 | 0.001 | 0.002 | 0.944 |
| p__WPS-2 | 0.002 | 0.006 | 0.001 | 0.003 | 0.000 | 0.000 | 0.001 | 0.002 | NA |
| p__Campilobacterota | 0.001 | 0.002 | 0.001 | 0.001 | 0.001 | 0.002 | 0.000 | 0.001 | 0.101 |
| p__Bdellovibrionota | 0.000 | 0.001 | 0.001 | 0.001 | 0.001 | 0.003 | 0.000 | 0.002 | 0.361 |

Note: E200; Fresh Amla fruit 200g/d; E400 , fresh Amla fruit 400 g/d; E600, fresh Amla fruit 600 g/d
