# Supplementary material for: The effect of Phyllanthus emblica (Amla) fruit supplementation on the rumen microbiota and its correlation with rumen fermentation in dairy cows
Source: Front Microbiol. 2024 May 13;15:1365681. doi: 10.3389/fmicb.2024.1365681 (PMC11128671; doi:10.3389/fmicb.2024.1365681)
Supplement: Supplementary file 4 [file Table_4.docx]

**Supplementary Table 4**: Mean average relative abundance and statistical difference of the genera in different groups

| Species name | E0-Mean (%) | E0-Sd (%) | E200-Mean (%) | E200-Sd (%) | E400-Mean (%) | E400-Sd (%) | E600-Mean (%) | E600-Sd (%) | P-value |
| --- | --- | --- | --- | --- | --- | --- | --- | --- | --- |
| g__Ruminococcus | 18.59 | 11.00 | 19.95 | 10.51 | 24.95 | 12.27 | 21.99 | 10.39 | 0.31 |
| g__Prevotella | 12.35 | 11.67 | 15.57 | 8.67 | 10.11 | 6.02 | 9.01 | 7.04 | 0.11 |
| g__NK4A214_group | 7.90 | 3.71 | 6.89 | 2.23 | 9.61 | 3.70 | 7.47 | 2.72 | 0.11 |
| g__norank_f__F082 | 4.73 | 3.45 | 7.19 | 6.17 | 6.13 | 5.93 | 8.51 | 8.39 | 0.18 |
| g__UCG-001 | 3.55 | 4.67 | 5.54 | 11.57 | 4.47 | 6.42 | 4.50 | 3.09 | 0.76 |
| g__Christensenellaceae_R-7_group | 5.18 | 2.44 | 4.45 | 1.84 | 4.07 | 2.80 | 4.11 | 1.52 | 0.20 |
| g__Rikenellaceae_RC9_gut_group | 2.42 | 2.31 | 4.17 | 2.70 | 1.92 | 1.83 | 3.96 | 4.51 | 0.04 |
| g__UCG-005 | 2.44 | 1.72 | 2.96 | 3.01 | 3.49 | 2.00 | 2.73 | 3.11 | 0.33 |
| g__norank_f__Bacteroidales_RF16_group | 2.37 | 2.54 | 2.85 | 2.79 | 1.38 | 1.37 | 3.67 | 2.27 | 0.01 |
| g__norank_f__Eubacterium_coprostanoligenes_group | 2.31 | 2.00 | 1.24 | 0.28 | 2.03 | 1.46 | 2.23 | 0.98 | 0.00 |
| g__CAG-352 | 2.67 | 4.68 | 1.21 | 1.23 | 1.54 | 1.51 | 2.21 | 1.81 | 0.14 |
| g__unclassified_f__Ruminococcaceae | 1.65 | 1.87 | 2.44 | 1.30 | 1.72 | 1.71 | 1.79 | 1.10 | 0.30 |
| g__unclassified_f__Prevotellaceae | 1.54 | 4.54 | 0.80 | 1.43 | 1.68 | 2.93 | 3.18 | 5.96 | 0.33 |
| g__norank_f__norank_o__Clostridia_UCG-014 | 1.54 | 1.56 | 1.15 | 0.79 | 1.95 | 1.16 | 2.08 | 1.63 | 0.08 |
| g__Candidatus_Saccharimonas | 1.37 | 1.04 | 1.56 | 0.98 | 1.51 | 1.56 | 1.87 | 0.84 | 0.33 |
| g__norank_f__UCG-010 | 1.21 | 0.88 | 1.85 | 0.99 | 1.30 | 1.34 | 1.33 | 0.79 | 0.18 |
| g__norank_f__Prevotellaceae | 2.09 | 3.66 | 0.74 | 0.54 | 1.25 | 1.28 | 1.14 | 1.03 | 0.06 |
| g__Lachnospiraceae_NK3A20_group | 1.24 | 1.77 | 1.31 | 2.24 | 1.48 | 2.88 | 0.95 | 1.57 | 0.90 |
| g__Prevotellaceae_UCG-003 | 1.41 | 2.62 | 0.98 | 0.40 | 0.66 | 0.39 | 1.27 | 0.87 | 0.02 |
| g__Bifidobacterium | 2.45 | 8.07 | 0.12 | 0.21 | 1.66 | 4.29 | 0.07 | 0.14 | 0.11 |
| g__norank_f__norank_o__RF39 | 0.97 | 0.89 | 0.81 | 0.57 | 1.43 | 1.57 | 0.99 | 0.70 | 0.50 |
| g__Prevotellaceae_UCG-001 | 1.21 | 1.69 | 0.99 | 1.17 | 0.67 | 0.49 | 0.68 | 0.35 | 0.18 |
| g__UCG-002 | 0.88 | 0.91 | 0.47 | 0.31 | 0.89 | 1.09 | 0.86 | 0.88 | 0.03 |
| g__norank_f__Muribaculaceae | 1.34 | 2.39 | 0.53 | 0.53 | 0.52 | 0.54 | 0.60 | 0.59 | 0.18 |
| g__Papillibacter | 0.94 | 0.74 | 0.74 | 0.47 | 0.53 | 0.55 | 0.73 | 0.48 | 0.16 |
| g__Kandleria | 0.72 | 1.57 | 0.30 | 0.57 | 0.58 | 1.17 | 1.15 | 3.56 | 0.37 |
| g__norank_f__norank_o__Rhodospirillales | 0.95 | 2.06 | 0.73 | 0.82 | 0.30 | 0.46 | 0.76 | 0.62 | 0.05 |
| g__norank_f__Ruminococcaceae | 0.45 | 0.55 | 0.51 | 0.68 | 1.19 | 2.09 | 0.33 | 0.33 | 0.32 |
| g__Olsenella | 1.07 | 3.79 | 0.14 | 0.21 | 1.18 | 3.95 | 0.09 | 0.12 | 0.19 |
| g__Treponema | 0.70 | 1.02 | 0.88 | 1.09 | 0.47 | 0.84 | 0.36 | 0.29 | 0.09 |
| g__Colidextribacter | 0.24 | 0.65 | 0.67 | 1.06 | 0.62 | 1.07 | 0.41 | 0.53 | 0.30 |
| g__norank_f__UCG-011 | 0.50 | 0.63 | 0.71 | 1.17 | 0.23 | 0.26 | 0.45 | 0.52 | 0.06 |
| g__Saccharofermentans | 0.60 | 0.92 | 0.40 | 0.46 | 0.49 | 0.65 | 0.23 | 0.20 | 0.04 |
| g__norank_f__norank_o__Bradymonadales | 0.69 | 1.39 | 0.02 | 0.02 | 0.03 | 0.04 | 0.95 | 2.40 | 0.00 |
| g__unclassified_f__Lachnospiraceae | 0.51 | 1.10 | 0.24 | 0.34 | 0.50 | 1.18 | 0.14 | 0.13 | 0.09 |
| g__U29-B03 | 0.38 | 1.05 | 0.55 | 0.82 | 0.20 | 0.32 | 0.16 | 0.21 | 0.20 |
| g__Ruminococcus_gauvreauii_group | 0.45 | 0.72 | 0.27 | 0.44 | 0.35 | 0.66 | 0.21 | 0.25 | 0.29 |
| g__Eubacterium_hallii_group | 0.41 | 0.71 | 0.28 | 0.34 | 0.24 | 0.39 | 0.23 | 0.33 | 0.53 |
| g__Acetitomaculum | 0.30 | 0.40 | 0.28 | 0.39 | 0.30 | 0.56 | 0.25 | 0.42 | 0.99 |
| g__norank_f__norank_o__Clostridia_vadinBB60_group | 0.25 | 0.54 | 0.31 | 0.25 | 0.16 | 0.24 | 0.19 | 0.15 | 0.26 |
| g__unclassified_f__Oscillospiraceae | 0.20 | 0.19 | 0.17 | 0.07 | 0.23 | 0.20 | 0.31 | 0.45 | 0.47 |
| g__Family_XIII_AD3011_group | 0.22 | 0.30 | 0.24 | 0.38 | 0.20 | 0.27 | 0.20 | 0.31 | 0.98 |
| g__unclassified_c__Clostridia | 0.31 | 0.57 | 0.15 | 0.13 | 0.17 | 0.15 | 0.22 | 0.17 | 0.25 |
| g__Prevotellaceae_UCG-004 | 0.29 | 0.47 | 0.12 | 0.10 | 0.27 | 0.40 | 0.15 | 0.09 | 0.08 |
| g__norank_f__norank_o__WCHB1-41 | 0.17 | 0.17 | 0.22 | 0.18 | 0.23 | 0.37 | 0.18 | 0.16 | 0.78 |
| g__Succinivibrionaceae_UCG-002 | 0.26 | 0.40 | 0.18 | 0.25 | 0.13 | 0.12 | 0.18 | 0.15 | 0.30 |
| g__norank_f__Oscillospiraceae | 0.18 | 0.28 | 0.16 | 0.13 | 0.23 | 0.14 | 0.16 | 0.12 | 0.43 |
| g__norank_f__Bacteroidales_UCG-001 | 0.07 | 0.15 | 0.48 | 0.83 | 0.02 | 0.02 | 0.07 | 0.11 | 0.01 |
| g__Monoglobus | 0.24 | 0.47 | 0.05 | 0.04 | 0.16 | 0.22 | 0.19 | 0.18 | 0.00 |
| g__Eubacterium_ventriosum_group | 0.09 | 0.09 | 0.21 | 0.21 | 0.18 | 0.32 | 0.13 | 0.09 | 0.11 |
| g__Lachnospiraceae_AC2044_group | 0.19 | 0.28 | 0.10 | 0.19 | 0.07 | 0.07 | 0.24 | 0.43 | 0.05 |
| g__Mogibacterium | 0.13 | 0.17 | 0.23 | 0.47 | 0.18 | 0.40 | 0.05 | 0.06 | 0.04 |
| g__Tyzzerella | 0.18 | 0.21 | 0.12 | 0.10 | 0.16 | 0.31 | 0.11 | 0.08 | 0.27 |
| g__norank_f__norank_o__Gastranaerophilales | 0.11 | 0.16 | 0.11 | 0.07 | 0.11 | 0.20 | 0.22 | 0.21 | 0.30 |
| g__unclassified_o__Oscillospirales | 0.16 | 0.20 | 0.11 | 0.07 | 0.16 | 0.15 | 0.10 | 0.07 | 0.24 |
| g__Succiniclasticum | 0.18 | 0.40 | 0.10 | 0.13 | 0.08 | 0.12 | 0.14 | 0.19 | 0.42 |
| g__UCG-004 | 0.12 | 0.17 | 0.21 | 0.27 | 0.05 | 0.07 | 0.10 | 0.11 | 0.05 |
| g__Eubacterium_ruminantium_group | 0.17 | 0.35 | 0.06 | 0.12 | 0.15 | 0.43 | 0.09 | 0.13 | 0.36 |
| g__Butyrivibrio | 0.16 | 0.22 | 0.12 | 0.16 | 0.05 | 0.07 | 0.12 | 0.18 | 0.02 |
| g__unclassified_f__Anaerovoracaceae | 0.17 | 0.32 | 0.08 | 0.10 | 0.09 | 0.19 | 0.08 | 0.09 | 0.39 |
| g__V9D2013_group | 0.03 | 0.02 | 0.20 | 0.20 | 0.15 | 0.22 | 0.05 | 0.04 | 0.00 |
| g__Prevotellaceae_YAB2003_group | 0.09 | 0.11 | 0.08 | 0.10 | 0.21 | 0.24 | 0.05 | 0.03 | 0.02 |
| g__Anaerovorax | 0.09 | 0.07 | 0.15 | 0.14 | 0.08 | 0.09 | 0.10 | 0.08 | 0.34 |
| g__Anaeroplasma | 0.07 | 0.09 | 0.21 | 0.30 | 0.03 | 0.04 | 0.06 | 0.07 | 0.03 |
| g__Lachnospiraceae_NK4A136_group | 0.04 | 0.10 | 0.04 | 0.05 | 0.23 | 0.61 | 0.03 | 0.04 | 0.58 |
| g__Fibrobacter | 0.07 | 0.13 | 0.18 | 0.40 | 0.04 | 0.04 | 0.04 | 0.03 | 0.21 |
| g__norank_f__p-251-o5 | 0.05 | 0.10 | 0.12 | 0.10 | 0.03 | 0.05 | 0.12 | 0.19 | 0.01 |
| g__Ruminobacter | 0.08 | 0.13 | 0.09 | 0.09 | 0.06 | 0.09 | 0.09 | 0.09 | 0.77 |
| g__DNF00809 | 0.11 | 0.18 | 0.10 | 0.18 | 0.07 | 0.12 | 0.04 | 0.06 | 0.11 |
| g__Eubacterium_nodatum_group | 0.10 | 0.15 | 0.09 | 0.12 | 0.08 | 0.12 | 0.04 | 0.06 | 0.11 |
| g__norank_f__Peptococcaceae | 0.08 | 0.07 | 0.06 | 0.06 | 0.09 | 0.07 | 0.09 | 0.06 | 0.42 |
| g__Alloprevotella | 0.06 | 0.10 | 0.03 | 0.06 | 0.09 | 0.16 | 0.10 | 0.15 | 0.22 |
| g__Pyramidobacter | 0.08 | 0.10 | 0.05 | 0.04 | 0.06 | 0.04 | 0.08 | 0.06 | 0.33 |
| g__norank_f__norank_o__Absconditabacteriales_SR1 | 0.09 | 0.12 | 0.03 | 0.02 | 0.09 | 0.09 | 0.05 | 0.05 | 0.01 |
| g__norank_f__Lachnospiraceae | 0.08 | 0.13 | 0.05 | 0.07 | 0.06 | 0.09 | 0.06 | 0.08 | 0.65 |
| g__Coprococcus | 0.07 | 0.10 | 0.05 | 0.07 | 0.05 | 0.07 | 0.07 | 0.11 | 0.60 |
| g__Marvinbryantia | 0.07 | 0.11 | 0.07 | 0.09 | 0.05 | 0.09 | 0.04 | 0.04 | 0.37 |
| g__Syntrophococcus | 0.09 | 0.20 | 0.05 | 0.07 | 0.05 | 0.07 | 0.03 | 0.04 | 0.15 |
| g__Prevotellaceae_NK3B31_group | 0.04 | 0.05 | 0.10 | 0.14 | 0.02 | 0.03 | 0.03 | 0.02 | 0.08 |
| g__norank_f__norank_o__Bacteroidales | 0.04 | 0.10 | 0.05 | 0.06 | 0.02 | 0.02 | 0.08 | 0.13 | 0.03 |
| g__Veillonellaceae_UCG-001 | 0.06 | 0.09 | 0.04 | 0.05 | 0.04 | 0.06 | 0.05 | 0.06 | 0.66 |
| g__Blautia | 0.07 | 0.10 | 0.05 | 0.07 | 0.04 | 0.05 | 0.04 | 0.05 | 0.51 |
| g__Sphaerochaeta | 0.06 | 0.13 | 0.07 | 0.11 | 0.04 | 0.11 | 0.02 | 0.02 | 0.14 |
| g__norank_f__norank_o__norank_c__norank_p__Armatimonadota | 0.02 | 0.06 | 0.01 | 0.01 | 0.10 | 0.25 | 0.05 | 0.08 | 0.16 |
| g__FD2005 | 0.09 | 0.34 | 0.02 | 0.01 | 0.03 | 0.04 | 0.05 | 0.06 | 0.11 |
| g__Succinivibrio | 0.15 | 0.85 | 0.01 | 0.02 | 0.02 | 0.03 | 0.00 | 0.00 | 0.13 |
| g__Bacillus | 0.05 | 0.06 | 0.03 | 0.04 | 0.03 | 0.05 | 0.07 | 0.08 | 0.26 |
| g__Lachnospiraceae_XPB1014_group | 0.05 | 0.08 | 0.07 | 0.18 | 0.02 | 0.04 | 0.03 | 0.06 | 0.18 |
| g__unclassified_k__norank_d__Bacteria | 0.03 | 0.04 | 0.04 | 0.04 | 0.02 | 0.04 | 0.08 | 0.17 | 0.35 |
| g__Defluviitaleaceae_UCG-011 | 0.04 | 0.06 | 0.07 | 0.11 | 0.03 | 0.05 | 0.03 | 0.03 | 0.41 |
| g__Erysipelotrichaceae_UCG-009 | 0.05 | 0.10 | 0.04 | 0.05 | 0.04 | 0.06 | 0.04 | 0.07 | 0.90 |
| g__norank_f__norank_o__norank_c__Clostridia | 0.03 | 0.05 | 0.04 | 0.08 | 0.06 | 0.12 | 0.04 | 0.05 | 0.80 |
| g__Prevotellaceae_Ga6A1_group | 0.04 | 0.06 | 0.04 | 0.05 | 0.05 | 0.14 | 0.02 | 0.03 | 0.39 |

Note: E200; Fresh Amla fruit 200g/d; E400 , fresh Amla fruit 400 g/d; E600, fresh Amla fruit 600 g/d
